# Spiromastixones Inhibit Foam Cell Formation via Regulation of Cholesterol Efflux and Uptake in RAW264.7 Macrophages

**DOI:** 10.3390/md13106352

**Published:** 2015-10-14

**Authors:** Chongming Wu, Ran Chen, Mingyue Liu, Dong Liu, Xin Li, Shuai Wang, Siwen Niu, Peng Guo, Wenhan Lin

**Affiliations:** 1 Pharmacology and Toxicology Research Center, Institute of Medicinal Plant Development, Chinese Academy of Medical Sciences, Peking Union Medical College, Beijing 100193, China; E-Mails: cmwu@implad.ac.cn (C.W.); cran_lxj@sina.com (R.C.); mingyueliu87@sohu.com (M.L.); aixiaoxin9333@sina.com (X.L.); zhuizhirun@163.com (S.W.); 2 State Key Laboratory of Natural and Biomimetic Drugs, Peking University, Beijing 100191, China; E-Mails: liudong_1982@126.com (D.L.); niusi123@126.com (S.N.)

**Keywords:** spiromastixones, *Spiromastix* sp., atherosclerosis, foam cell, PPARγ, ABCA1/G1, CD36

## Abstract

Bioassay-guided evaluation shows that a deep sea-derived fungus, *Spiromastix* sp. MCCC 3A00308, possesses lipid-lowering activity. Chromatographic separation of a culture broth resulted in the isolation of 15 known depsidone-based analogues, labeled spiromastixones A–O (**1**–**15**). Each of these compounds was tested for its ability to inhibit oxidized low-density lipoprotein (oxLDL)-induced foam cell formation in RAW264.7 macrophages. Spiromastixones **6**–**8** and **12**–**14** significantly decreased oxLDL-induced lipid over-accumulation, reduced cell surface area, and reduced intracellular cholesterol concentration. Of these compounds, spiromastixones **6** and **14** exerted the strongest inhibitory effects. Spiromastixones **6** and **14** dramatically inhibited cholesterol uptake and stimulated cholesterol efflux to apolipoprotein A1 (ApoA1) and high-density lipoprotein (HDL) in RAW264.7 macrophages. Mechanistic investigation indicated that spiromastixones **6**, **7**, **12** and **14** significantly up-regulated the mRNA levels of ATP-binding cassette sub-family A1 (ABCA1) and down-regulated those of scavenger receptor CD36, while the transcription of ATP-binding cassette sub-family A1 (ABCG1) and proliferator-activated receptor gamma (PPARγ) were selectively up-regulated by **6** and **14**. A transactivation reporter assay revealed that spiromastixones **6** and **14** remarkably enhanced the transcriptional activity of PPARγ. These results suggest that spiromastixones inhibit foam cell formation through upregulation of PPARγ and ABCA1/G1 and downregulation of CD36, indicating that spiromastixones **6** and **14** are promising lead compounds for further development as anti-atherogenic agents.

## 1. Introduction

Atherosclerosis (AS) is the primary risk factor associated with coronary artery disease, is the leading cause of morbidity and mortality in developed countries, and is becoming increasingly prevalent in developing countries [1]. Atherosclerosis is a progressive disease that is characterized by the formation and accumulation of lipid plaques in the arteries and by inflammatory responses, which together result in insufficient blood supply to organs and tissues. About half of all cardiovascular disease deaths are caused by atherosclerosis [2,3]. According to the current understanding of the cellular and molecular mechanisms underlying atherogenesis, the most important event in the development of atherosclerosis is the accumulation of extracellular and intracellular lipids in the arterial intima caused by low-density lipoprotein (LDL) [4,5]. The resultant lipid-laden cells are known as foam cells and are an essential feature and key attribute of atherosclerotic lesions [4]. Foam cell formation is associated with increased macrophage cholesterol levels and results from imbalanced lipid efflux and influx [4]. Foam cell formation is a main determinant of the occurrence of atherosclerotic lesions. In atherosclerotic lesions, macrophages express scavenger receptors on their plasma membranes; uptake oxidized LDL, which is deposited into blood vessel walls; and develop into foam cells [6]. Foam cells secrete various inflammatory cytokines and accelerate the development of atherosclerosis [6]. For instance, scavenger receptors CD36, SR-A1 and SR-A2 bind to and uptake excess oxidized low-density lipoprotein (oxLDL) into macrophages [7], leading to the accumulation of excess cholesterol, which is toxic to cells. A group of ATP-binding cassette (ABC) transporters, including ABCA1 and ABCG1, has been shown to play a critical role in the reverse cholesterol transport (RCT) pathway by mediating the translocation of cholesterol across cellular bilayer membranes [8,9,10]. ABCA1 promotes the efflux of cholesterol to lipid-poor apolipoproteins, such as apolipoprotein A1 (apoA1), while ABCG1 plays a critical role in mediating cholesterol efflux to high-density lipoprotein (HDL) [10,11,12]. The expression of ABCA1 and ABCG1 is, to some extent, regulated by proliferator-activated receptor gamma (PPARγ)-dependent and liver X receptor alpha (LXRα)-dependent pathways, respectively [13,14]. Recent studies have shown that agonists of peroxisome PPARγ, such as rosiglitazone, can stimulate cholesterol efflux by upregulating the expression of ABCA1, which may or may not be dependent on LXRα [14]. Therefore, preventing foam cell formation via the inhibition of cholesterol influx and the promotion of cholesterol efflux provides an option for the treatment of atherogenesis.

Currently, statins and other commercially available drugs have demonstrated that it is possible to prevent and correct atherosclerosis. However, it has become clear that the widespread use of synthetic drugs, such as statins, for the prevention of atherosclerosis in its early stages is unlikely due to their narrow indications for prescription and severity of side effects [15,16,17]. However, natural products or products of natural origin may be considered promising drugs for anti-atherosclerotic therapy. Numerous natural products have been shown to possess not only anti-atherogenic properties but also pro-atherogenic or atherogenic-neutral properties [18,19,20,21]. Among the known anti-atherogenic natural products, the most effective is garlic [22,23]. In an attempt to discover natural anti-atherogenic products in marine-derived microorganisms, a cell model-based bioassay was performed. The results demonstrated that a deep-sea *Spiromastix* sp. fungus can reduce lipid accumulation. Chromatographic separation of an active lipid-lowering ethyl acetate (EtOAc) fraction from the fungus led to the isolation of fifteen depsidone-based analogues, designated spiromastixones A–O. In this paper, we report the inhibitory effects of spiromastixones A–O on oxLDL-induced foam cell formation and detail the potential mechanisms of this inhibition in RAW264.7 macrophages.

## 2. Results and Discussion

### 2.1. Structural Characterization of Spiromastixones

**Figure 1:**
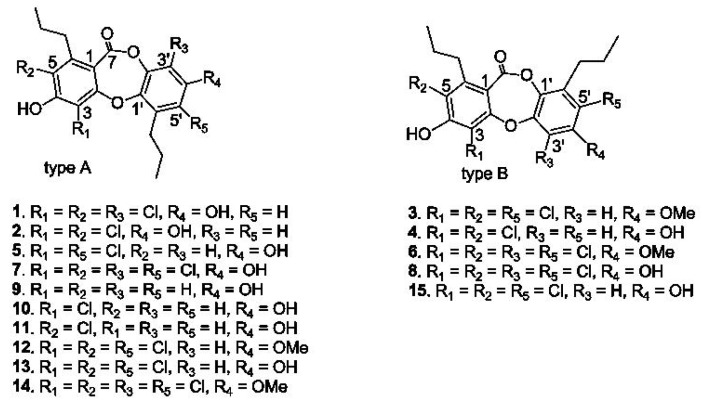
Structures of spiromastixones **1**–**15**.

Chemical examination of a fermentation broth of a deep-sea *Spiromastix* sp. fungus resulted in the isolation of fifteen depsidone-based analogues, termed spiromastixones A–O (**1**–**15**) (Figure 1), which were previously isolated from the same fungus [24]. Their structures were elucidated on the basis of extensive nuclear magnetic resonance (NMR) and mass spectroscopic analyses in association with chemical conversion. Spiromastixones A–O were classified into two subtypes based on the orientation of ring C relative to ring A. The *n*-propyl substituents on rings A and C are rarely observed in natural products. Most analogues contain substitutions of various numbers of chlorine atoms [24].

### 2.2. Spiromastixones Decrease oxLDL-Induced Lipid Overaccumulation in RAW264.7 Cells

**Figure 2:**
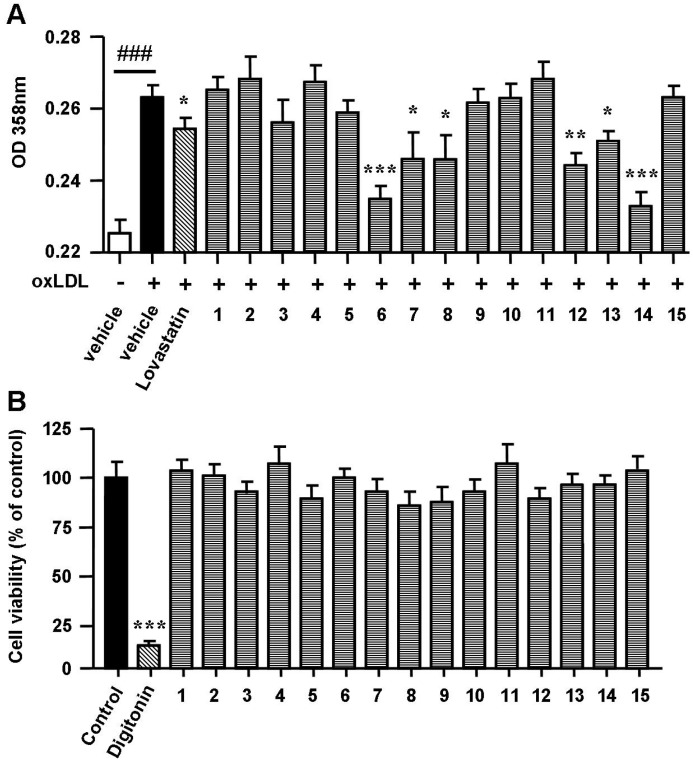
Effects of spiromastixones **1**–**15** on oxidized low-density lipoprotein (oxLDL)-induced lipid accumulation (**A**) and on cell viability (**B**) in RAW264.7 macrophages. Cells were incubated with Dulbecco’s modified Eagle’s medium (DMEM) + oxLDL (50 μg/mL) for 12 h and then treated with 10 μM of each of the indicated compounds or lovastatin (as a positive control) in DMEM + 100 μM oxLDL, with DMEM alone (as a blank) or with DMEM + 50 μg/mL oxLDL (as a negative control) for an additional 12 h. Concentrations of neutral lipids were determined by spectrophotometry at 358 nm after Oil Red O staining. Cytotoxicity was assessed by the MTT assay at 40 μM. Digitonin (40 μM) was used as a positive control. The values depicted are the means ± SEM of at least three experiments. ### *p* < 0.001, oxLDL *vs.* Blank; * *p* < 0.05, ** *p* < 0.01, *** *p* < 0.001, test group *vs.* oxLDL group.

The uptake of oxLDL by macrophages induces foam cell formation and promotes the development of atherosclerosis [12]. To determine the effects of spiromastixones on oxLDL-induced foam cell formation, macrophages were exposed to oxLDL, after which Oil Red O staining was performed. RAW264.7 macrophages were incubated with oxLDL (50 mg/mL) for 24 h. The addition of oxLDL to the culture medium induced foam cell formation, as shown by the increase in cytoplasmic lipid accumulation (Figure 2A). Supplementation of spiromastixones **6**, **7**, **8**, **12**, **13**, and **14**, as well as of lovastatin (10 μM), markedly decreased oxLDL-mediated neutral lipid accumulation in RAW264.7 cells (Figure 2A). As determined by the 3-(4,5-dimethylthiazol-2-yl)-2,5-diphenyltetrazolium bromide (MTT) assay, these compounds did not decrease cell viability in RAW264.7 macrophages at concentrations of 1 to 40 μM (Figure 2B), indicating that their inhibitory effects on cellular lipid accumulation were not due to cytotoxicity.

**Figure 3:**
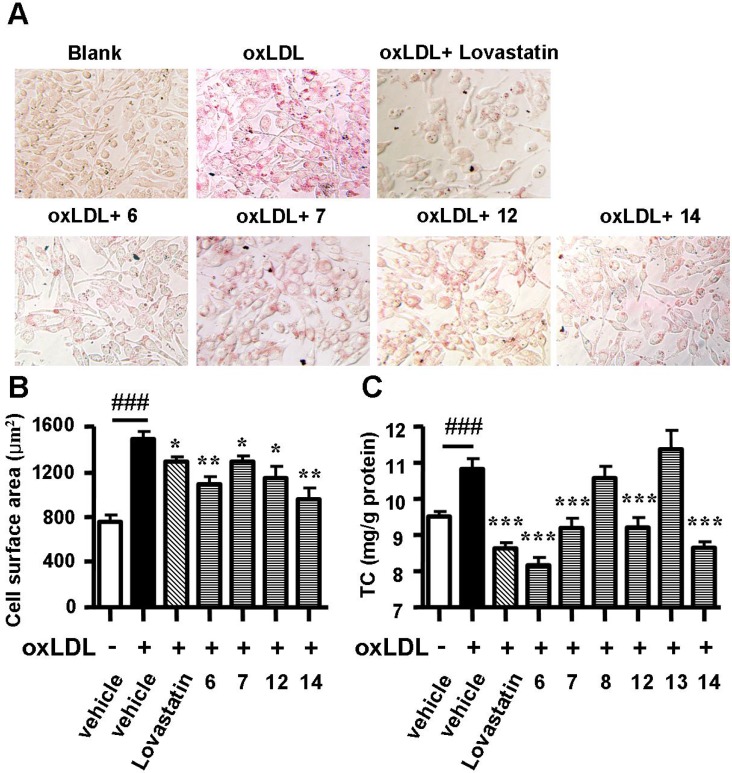
Effects of spiromastixones **6**, **7**, **8**, **12**, **13**, and **14** on oxLDL-induced foam cell formation in RAW264.7 macrophages. (**A**) Representative pictures taken after Oil Red O staining; (**B**) cell surface areas as measured by Image-Pro Plus; (**C**) intracellular total cholesterol (TC) levels. The values shown are the means ± SEM of at least three experiments. ### *p* < 0.001, oxLDL *vs.* Blank; * *p* < 0.05, ** *p* < 0.01, *** *p* < 0.001, test group *vs.* oxLDL group.

Cell surface enlargement and intracellular cholesterol accumulation are two signs of the formation of a foam cell. Therefore, a second round of evaluation assays consisting of photography after Oil Red O staining and total intracellular cholesterol quantification were performed on the six positive compounds. Spiromastixones **6**, **7**, **12** and **14** largely alleviated neutral lipid accumulation (Figure 3A), reduced the cell surface area (Figure 3B) and significantly decreased the intracellular total cholesterol concentration (Figure 3C), while spiromastixones **8** and **13** showed no effect (Figure 3B). The inhibitory efficiencies of spiromastixones **6** and **14** were comparable to that of lovastatin at the same dose. These results suggested that spiromastixones **6**, **7**, **12** and **14** are adequate in preventing the formation of foam cells induced by oxLDL in RAW264.7 macrophages.

### 2.3. Spiromastixones Inhibit Cholesterol Uptake by RAW264.7 Macrophages

**Figure 4:**
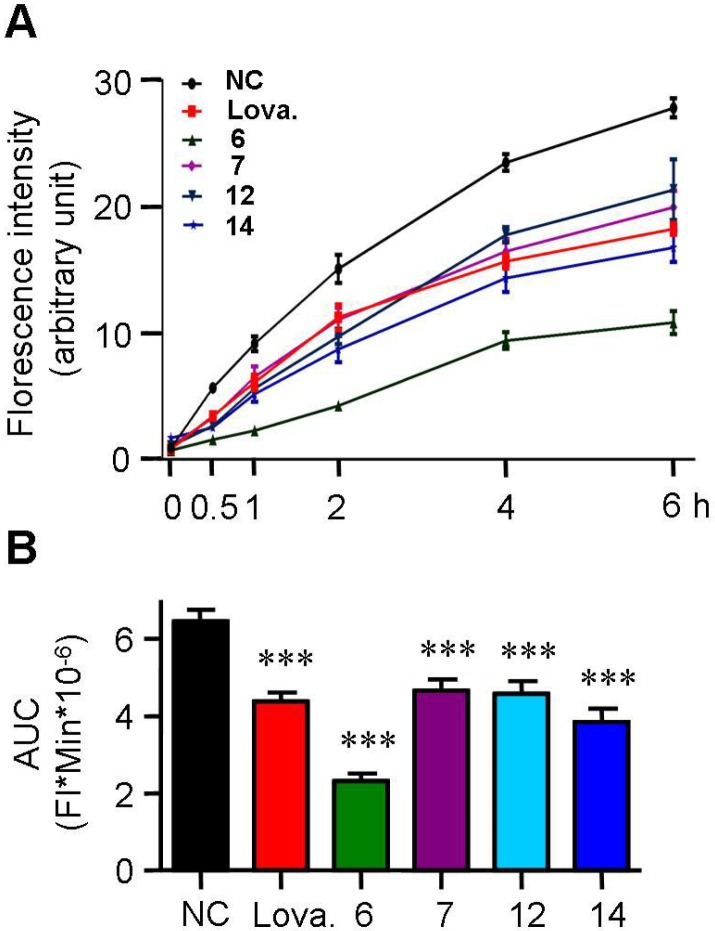
Effects of spiromastixones **6**, **7**, **12**, and **14** on cholesterol uptake by RAW264.7 macrophages. (**A**) Time-dependent cholesterol uptake curves indicated by 7-nitrobenz-2-oxa-1,3-diazol-4-yl (NBD)-cholesterol; (**B**) area under the curve (AUC). The concentrations of spiromastixones **6**, **7**, **12**, and **14**, as well as lovastatin (Lova.), were 10 μM. The values shown are the means ± SEM of at least three experiments. *** *p* < 0.001 *vs.* the negative control (NC) group.

The formation of foam cells is typically caused by either uncontrolled uptake of cholesterol or impaired cholesterol efflux. We evaluated the effects of active spiromastixones on cholesterol influx using 25-{*N*-[(7-nitrobenz-2-oxa-1,3-diazol-4-yl)-methyl]amino}-27-norcholesterol (25-NBD cholesterol) as an indicator. As shown in Figure 4A, spiromastixones **6**, **7**, **12** and **14** inhibited cholesterol uptake by RAW264.7 macrophages in a dose-dependent manner, which can partially account for the decrease in the intracellular cholesterol levels (Figure 3C). Quantification of the area under the curve of the fluorescence intensity (FI) shows that these compounds potently inhibited cholesterol uptake with efficacies either comparable to or more pronounced than that of lovastatin (Figure 4B). These results suggest that spiromastixones **6**, **7**, **12** and **14** effectively inhibit cholesterol uptake by macrophages.

### 2.4. Spiromastixones Promote Cholesterol Efflux from RAW264.7 Macrophages

**Figure 5:**
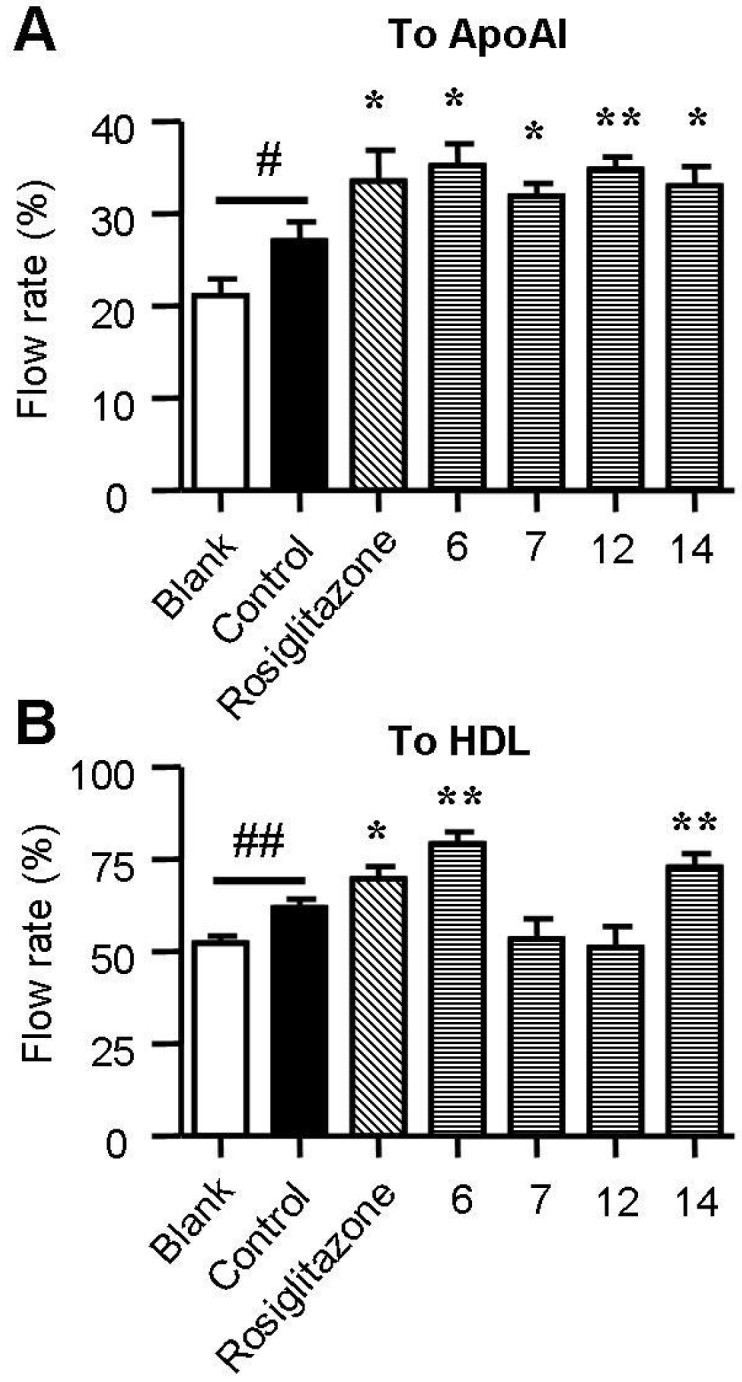
Effects of spiromastixones **6**, **7**, **12**, and **14** on NBD-cholesterol efflux to ApoA1 (**A**) and HDL (**B**) in RAW264.7 macrophages. Cells were equilibrated with NBD-cholesterol for 12 h and then incubated in serum-free DMEM medium containing ApoA1 or HDL and 10 μM of the indicated compounds or rosiglitazone (as a positive control) for 6 h. Cholesterol efflux was expressed as the percentage of fluorescence in the medium relative to the total amounts of fluorescence detected in the cells and the medium. The values shown are the means ± SEM of at least three experiments. # *p* < 0.05, ## *p* < 0.01, Control *vs.* Blank; * *p* < 0.05, ** *p* < 0.01, test group *vs.* control.

Cholesterol efflux from macrophages is the first and potentially most important step in reverse cholesterol transport (RCT), which increasing evidence has shown to be an effective anti-atherogenic strategy [12,25]. ApoA1 and HDL are two key receptors of cholesterol efflux. A 25-NBD cholesterol-based cholesterol efflux assay was used to assess the effect of chrysin on cholesterol efflux in RAW264.7 macrophages. As shown in Figure 5, supplementation with rosiglitazone, a commercially available cholesterol efflux stimulator, greatly increased ApoA1-mediated and HDL-mediated cholesterol efflux. Treatment with spiromastixones **6**, **7**, **12** and **14** significantly increased ApoA1-mediated cholesterol efflux with an efficiency comparable to that of rosiglitazone (Figure 5A). Spiromastixones **6** and **14** also stimulated HDL-mediated cholesterol efflux and produced higher cholesterol efflux rates than that of rosiglitazone (Figure 5B). These results demonstrated that spiromastixones from *Spiromastix* sp. can inhibit macrophage-derived foam cell formation by inhibiting cholesterol influx and promoting cholesterol efflux.

### 2.5. Spiromastixones Alter mRNA Levels of Cholesterol Efflux/Influx-Modulating Genes and PPARγ Transcriptional Activity

**Figure 6:**
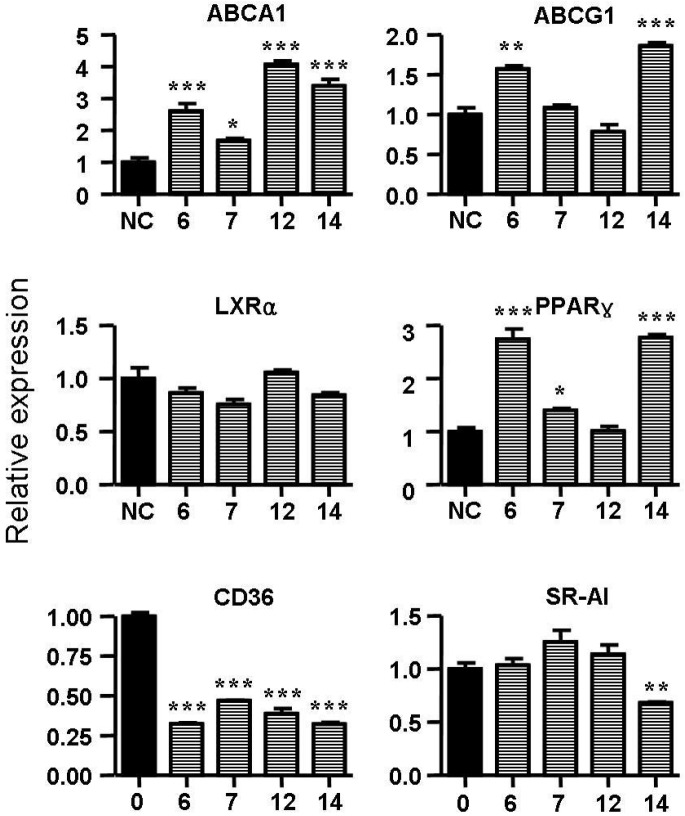
Effects of spiromastixones **6**, **7**, **12**, and **14** on the mRNA levels of PPARγ, LXRα, ABCA1, ABCG1, CD36 and scavenger receptor-1 (SR-1) in RAW264.7 cells. Real-time PCR was conducted with gene-specific oligonucleotide primers. The amplification of β-actin served as an internal control. The values shown are the means ± SEM of at least three experiments. * *p* < 0.05, ** *p* < 0.01, *** *p* < 0.001 *vs.* control.

**Figure 7:**
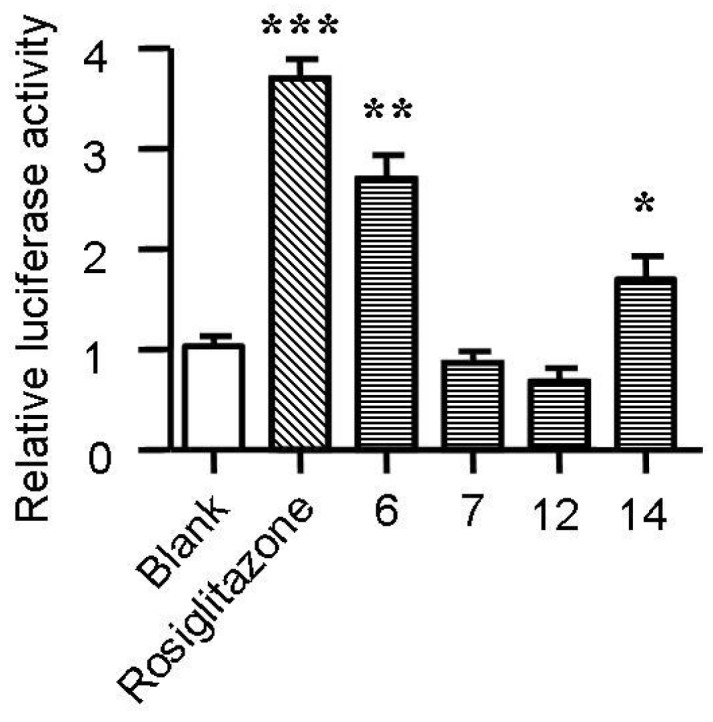
Effects of spiromastixones **6**, **7**, **12**, and **14** on the transcriptional activity of PPARγ. The transcriptional activity of PPARγ was assessed by a transactivation reporter assay in 293T cells. The concentrations of spiromastixones **6**, **7**, **12**, and **14**, as well as of rosiglitazone, were each 10 μM. The values shown are the means ± SEM of at least three experiments. * *p* < 0.05, ** *p* < 0.01, *** *p* < 0.001 *vs.* control.

Cholesterol flux in macrophages is tightly regulated by several genes. ABCA1, ABCG1, LXRα and PPARγ are key regulators of cholesterol efflux, while CD36, SR-A1 and SR-A2 are critical scavenger receptors involved in cholesterol uptake [25,26]. Treatment with spiromastixones **6**, **7**, **12** and **14** (10 μM) significantly decreased CD36 transcription and increased the mRNA level of ABCA1 (Figure 6). The spiromastixone-mediated regulation of these genes could explain their inhibition of cholesterol uptake and promotion of ApoA1-mediated cholesterol efflux. Spiromastixones **6** and **14** also increased the transcription of ABCG1 and PPARγ, which may explain their roles in promoting HDL-mediated cholesterol efflux. A luciferase assay showed that spiromastixones **6** and **14** significantly elevated the transcriptional activity of PPARγ (Figure 7), suggesting that these two spiromastixones may stimulate cholesterol efflux via upregulation of the PPARγ-ABCA1/G1 pathway. The differential regulation of PPARγ and ABCG1 may explain the different effects of spiromastixones **6** and **14** and compounds **7** and **12** on cholesterol efflux to HDL.

### 2.6. Structure-Activity Relationship of Spiromastixones

Analyses of structure-activity relationships revealed that the inhibitory effects of oxLDL-mediated neutral lipid accumulation in RAW264.7 cells were directly induced by substitutions in molecular scaffolds, whereas ring fusion modifications, such as those observed in types A and B, weakly affected their activities. Compounds **6** and **14**, possessing a 3,5,3′,5′-tetrachlorinated and a 4′-methoxylated substitution, showed the most potent activities compared to the relative analogues. Compounds **7** and **8**, each with a hydroxyl group replacing the methoxy group at C-4′, reduced the effect, indicating that the methoxy group at C-4′ enhances their activities. This conclusion was also supported by the increased effects of compounds **3** and **12** compared to those produced by compounds **13** and **8**. The latter compounds differed from **3** and **12** solely by the substitution of a hydroxyl group at C-4′ instead of a methoxy group. In addition, the analogues with one substituted chlorine atom (**10**, **11**), two chlorine atoms (**2**, **5**), and no chlorine atoms (**9**) showed weaker activities compared to those with three or four chlorine atoms (**6**–**8**, **12**–**14**). Thus, we conclude that the most favorable spiromastixones are 3,3′,5,5′-tetrachloro-4′-methoxy analogues, such as **6** and **14**. These compounds displayed greater lipid-lowering effects compared to the positive control lovastatin (Figure 2). These data suggest that both compounds represent promising leads for the development of new anti-atherogenic agents.

## 3. Experimental Section

### 3.1. Materials and Reagents

25-[*N*-[(7-nitrobenz-2-oxa-1,3-diazol-4-yl)-methyl]amino]-27-norcholesterol (25-NBD cholesterol), MTT, digitonin, lovastatin, rosiglitazone, Oil Red O and Dulbecco’s modified Eagle’s medium (DMEM) were procured from Sigma-Aldrich, Inc. (St. Louis, MO, USA). An intracellular cholesterol assay kit was purchased from Jian Cheng Biotechnology Company (Nanjing, China). Human oxLDL, ApoA1 and HDL were obtained from Yiyuan Biotechnologies (Guangzhou, China). A total RNA extraction reagent (RNAiso Plus), a PrimeScript RT reagent kit, and a SYBR-Green PCR kit were purchased from Transgene Biotech, Inc. (Beijing, China).

### 3.2. Cell Culture

RAW264.7 cells, which originated from the American Type Culture Collection (ATCC) (Manassas, VA, USA), were obtained from the Peking Union Medical College. The cells were maintained in DMEM medium (Gibco, Grand Island, NY, USA) supplemented with 10% fetal bovine serum (Gibco), penicillin (100 U/mL) and streptomycin (100 μg/mL) at 37 °C in 5% CO_2_. After being grown to 70%–80% confluence, the cells were incubated in DMEM supplemented with oxLDL (50 mg/mL, Xiesheng Biotechnologies, Beijing, China) and 10 μM of each of the experimental compounds individually for 12 h. The vehicle used to deliver these compounds was dimethyl sulphoxide (DMSO) at a final concentration of 0.1%. Subsequently, the cells were subjected to Oil Red O staining or total cholesterol determination as described previously [27].

### 3.3. Oil Red O Staining

Lipid staining was assessed histologically using Oil Red O staining. Treated RAW264.7 cells were incubated with oxLDL (50 mg/mL) in medium containing lipoprotein-deficient human serum for 24 h. The cells were then fixed with 4% w/v paraformaldehyde (30 min, room temperature) and stained with filtered Oil Red O solution (60 min, room temperature). The staining was evaluated by both microscopic examination (Olympus, Tokyo, Japan) and spectrophotometry at 358 nm.

### 3.4. Measurement of Cholesterol in Macrophages

Concentrations of intracellular cholesterol were determined by kits as previously reported [28]. Protein pellets were solubilized in 1 M NaOH (Sinopharm Chemical Reagent Co., Ltd, Shanghai, China), and the protein concentration was determined using a BCA Protein Assay (Thermo Fisher Scientific Inc., Rockford, IL, USA).

### 3.5. 25-NBD Cholesterol Uptake Assay

Cholesterol uptake assays were performed using 25-NBD cholesterol in RAW264.7 macrophages. The cells were plated in 96-well clear-bottom black plates (Costar, Corning Inc., Corning, NY, USA) at 4 × 10^4^ cells/well. Six hours later, the medium was removed, and the cells were labeled with 25-NBD cholesterol (5 µg/mL) in aliquots of serum-free DMEM individually containing 10 μM of each of the experimental compounds or an equal volume of DMSO for indicated time. Then, the cells were washed twice with phosphate buffered saline (PBS), and the amounts of cholesterol in the cells were measured using a Tecan Infinite M1000Pro Microplate Reader (TECAN Group Ltd., Shanghai, China; excitation 485 nm, emission 535 nm). Each uptake assay was performed in duplicate in three experiments.

### 3.6. Cholesterol Efflux Assay

RAW264.7 cells were equilibrated with NBD-cholesterol (1 μg/mL) for 12 h. The NBD-cholesterol-labeled cells were washed with PBS and incubated in serum-free DMEM medium containing 50 μg/mL HDL or ApoA1 and 10 μM of each of the experimental compounds individually for 6 h. Fluorescence-labeled cholesterol released from the cells into the medium was measured with a Tecan Infinite M1000Pro Microplate Reader (TECAN Group Ltd., Shanghai, China). Cholesterol efflux was expressed as a percentage of fluorescence in the medium relative to the total amounts of fluorescence detected in the cells and the medium. Each experiment was performed in triplicate with 3 replicates each time.

### 3.7. Real-Time Quantitative PCR

Total RNA extraction, cDNA synthesis and quantitative PCR assays were performed as described previously [29]. At least three independent biological replicates were performed to verify the reproducibility of the data. The gene-specific primers used for quantitative PCR are listed in Table 1.

**Table 1:** Primers used in real-time quantitative PCR analysis.

Name	Forward (5′-3′)	Reverse (5′-3′)
PPARγ	GCAGCTACTGCATGTGATCAAGA	GTCAGCGGGTGGGACTTTC
LXRα	AGGAGTGTCGACTTCGCAAA	CTCTTCTTGCCGCTTCAGTTT
ABCA1	CCCAGAGCAAAAAGGGACTC	GGTCATCATCACTTTGGTCCTTG
ABCG1	CAAGACCCTTTTGAAAGGGATCTC	GCCAGAATATTCATGAGTGTGGAC
CD36	CAAGCTCCTTGGCATGGTAGA	TGGATTTGCAAGCACAATATGAA
SR-A1	TTAAAGGTGATCGGGGACAAA	CAACCAGTCGAACTGTCTTAAG
β-actin	CCTGGCACCCAGCACAAT	GCCGATCCACACACGGAGTACT

### 3.8. Measurement of PPARγ Promoter Activity

A transactivation reporter assay in 293T cells was performed as previously described [30]. Briefly, cells were transiently transfected with a PPARγ expression vector and a DR-1 luciferase reporter vector. At 6 h after transfection, the transfection mixture was replaced with fresh medium containing the appropriate agonist. Luciferase assays were performed after 24 h using a luciferase assay kit (Promega, Beijing, China) according to the manufacturer’s instructions.

### 3.9. Cell Viability Assay

Cell viability was examined using an MTT assay. RAW264.7 macrophages in 96-well culture plates were treated with 1, 5, 10, and 40 μM of spiromastixones **1**–**15** or with 40 μM digitonin as a cytotoxic control. The cells were incubated for 12 h, and MTT reagent (5 mg/mL) was added to each well. After 2 h, the medium was removed and cells were lysed in 200 μL of DMSO. The absorbance at 565 nm was measured using a microplate reader (TECAN Group Ltd., Shanghai, China).

### 3.10. Statistical Analyses

The data are presented as the mean ± SEM. Differences were assessed by one-way analysis of variance (ANOVA) followed by Dunnett’s *post hoc* test. A probability level (*p*) of 0.05 was considered significant. SPSS 17.0 for Windows (SPSS, Chicago, IL, USA) was used for statistical analysis.

## 4. Conclusions

The present work detailed a group of natural products with unique scaffolds, namely spiromastixones, that efficiently inhibited foam cell formation in RAW264.7 macrophages. Analyses of their structure-activity relationships revealed that the inhibitory effects of spiromastixones are directly related to specific substitutions, particularly the number of chlorine atoms and the methoxy group at C-4′, which play an important role in reducing lipid accumulation and foam cell formation. Mechanistic investigation revealed that spiromastixones, such as compounds **6** and **14**, promoted cholesterol efflux through upregulation of the PPARγ-ABCA1/G1 pathway and inhibited cholesterol uptake via downregulation of the scavenger receptors CD36 and SR-A1. These findings suggest that spiromastixones **6** and **14** are promising leads for the development of a new type of anti-atherosclerotic agent. These findings also support the idea that marine-derived microorganisms are a promising potential source for the discovery of new drugs to treat AS.
